# New Advances in the Understanding of Proteases as Diagnostic and Pharmaceutical Targets in Homeostatic and Pathologic Conditions

**DOI:** 10.3390/pharmaceutics14071516

**Published:** 2022-07-21

**Authors:** Andrey A. Zamyatnin, Alessandro Parodi

**Affiliations:** 1 Biotechnology Department, Sirius University of Science and Technology, Olympic Avenue, 1, 354349 Sirius, Russia; zamyat@belozersky.msu.ru; 2 Institute of Molecular Medicine, Sechenov First Moscow State Medical University, 119991 Moscow, Russia; 3 Belozersky Institute of Physico-Chemical Biology, Lomonosov Moscow State University, 119992 Moscow, Russia; 4 Faculty of Health and Medical Sciences, University of Surrey, Guildford GU2 7X, UK

Protease biology represents a hot topic in biomedical research because of their pivotal role in regulating cell and tissue homeostasis, regeneration and pathogenesis. Their activity regulates vital processes both within the cell cytoplasm and in the extracellular environment. However, the high number of proteolytic enzymes (proteases represent about 2% of the genome) and their overlapping specificity make their investigation very complicated. An additional obstacle to their deep comprehension and targetability in terms of pharmaceutical development is represented by their heterogeneity and biological regulation determined by their cellular or extracellular location, activation state and interaction with endogenous inhibitors. The goal of this Special Issue was to collect recent advances in protease investigation from both the biological and the therapeutic standpoint.

As highlighted by many works, including the ones belonging to this collection, protease activity is finely regulated at the post-transcriptional level and therefore traditional gene and protein expression evaluation can provide only a rough indication of their contribution to a pathologic or physiologic process. For this reason, Howng et al. [1] dedicated considerable efforts to developing a novel test to evaluate tissue protease activity via zymography. This test could improve the sensitivity of current zymography approaches often affected by unspecific protein degradation during sample preparation. The test proposed in this Special Issue is based on the use of Probody^®^ therapeutic(s) (Pb-Tx) technology that consists in tumor targeting antibodies in which the antigen-binding domain is sterically inhibited by a peptide linked through specific protease-cleavable linkers. This technology was conceived to respond to the tumor proteolytic environment and acting only in the sick tissue, where the peptide removal was likely to occur. Pb-Tx was successfully proven to work as a diagnostic tool by labeling the antibodies with a fluorescent reporter and performing the analysis with capillary electrophoresis to detect activated antibodies after incubation with the tissue. The specificity of the analysis could be further refined by the use of inhibitors to discern between proteases with overlapping activity. In addition to their anticancer activity, these antibodies provided information about the proteolytic activity in preclinical and clinical cancer tissues, including human plasma. 

The potential role of developing theranostics based on endogenous proteases has been addressed also in the review of Sotiropoulou et al. [2]. They highlighted the potentialities of activity-based probes (ABP) in determining active proteolytic elements in a biological environment and their use as therapeutic tools, since they often work as proteolytic inhibitors. These molecules were extensively used to target different families of proteases, including kallikrein-related peptidases, cathepsins and neutrophils elastases. From the therapeutic point of view, ABP can treat many conditions varying from atherosclerosis to cancer. As diagnostic elements for proteolytic activity evaluation, they can be coupled with different reporters as fluorescent molecules and iodine for CT-scan analysis. In this context, ABP were engineered as polymeric dendrimers, highlighting the advantages that a drug delivery system could provide to target these molecules.

Hanning et al. [3] demonstrated the role of proteases in the pathophysiology of inflammatory bowel disease and irritable bowel syndrome. In particular, they showed an increase in trypsin-like and elastase-like activity in the feces of animals induced for colitis, in comparison with healthy controls. They showed that the local administration of the serine protease inhibitor UAMC-00050 could mitigate the development of the tissue inflammation favoring the resolving of the disease, while mitigating tissue damaged. 

On the other hand, proteases of exogenous sources could represent a valid tool for developing novel concepts of therapeutics. The serine protease D10 isolated from *Bacillus Subtilis* was tested in combination with the antimicrobial agent silver sulfadiazine to mitigate chemical burn wound damage. In this work, Al-Dhuayan et al. [4] demonstrated that the topical application of these treatments could accelerate skin regeneration with normalization of the different tissue elements, including dermis granulated follicles, fibroblasts and blood vessels and hypodermis ultrastructure. The beneficial action of the D10 protease was attributed to its ability to clear necrotic debris and target fibrin proteins and collagen fibers of the damaged tissue, accelerating the new deposition and recycling of these damage proteins while favoring local blood capillary growth. 

Finally, Mijanovic and Petushkova et al. [5] generated a comprehensive review regarding the role of Cathepsin D in physiological and pathological conditions, highlighting the importance of investigating single enzymes in different contexts. This enzyme is involved in important processes such as autophagy, protein recycling and activation of growth factors and other enzymes. Its downregulation is associated with neurological and lysosomal disorders. On the other hand, its overexpression and extra-lysosomal location were associated with diabetes and tumor growth, spreading and angiogenesis. For these reasons, this enzyme represents an optimal diagnostic and pharmaceutical target. 

In conclusion, protease investigation is gaining increasing interest by the scientific community because of their pleiotropic role in many diseases. We hope this collection has helped in understanding the importance and the potential of investigating proteases as pivotal players in our body and therapeutic agents/targets. More efforts should be dedicated towards developing novel tests to identify the proteolytic profile in different pathologies and the regulation mechanisms modulating protein degradation. Finally, it is worth mentioning that this field offers different avenues of patenting and biotech business, since the discovery of more effective and specific inhibitors and the delivery of pro- or anti-proteolytic agents is needed both for understanding physiological processes and for therapeutic and diagnostic purposes.
